# Supplementary material for: Evolution of tropical cyclone genesis regions during the Cenozoic era
Source: Nat Commun. 2019 Jul 12;10:3076. doi: 10.1038/s41467-019-11110-2 (PMC6625981; doi:10.1038/s41467-019-11110-2)
Supplement: Supplementary file 1 — Supplementary Information [file 41467_2019_11110_MOESM1_ESM.pdf]

Supplementary Information

Evolution of tropical cyclone genesis regions during the Cenozoic era

Yan et al.

Supplementary Figures

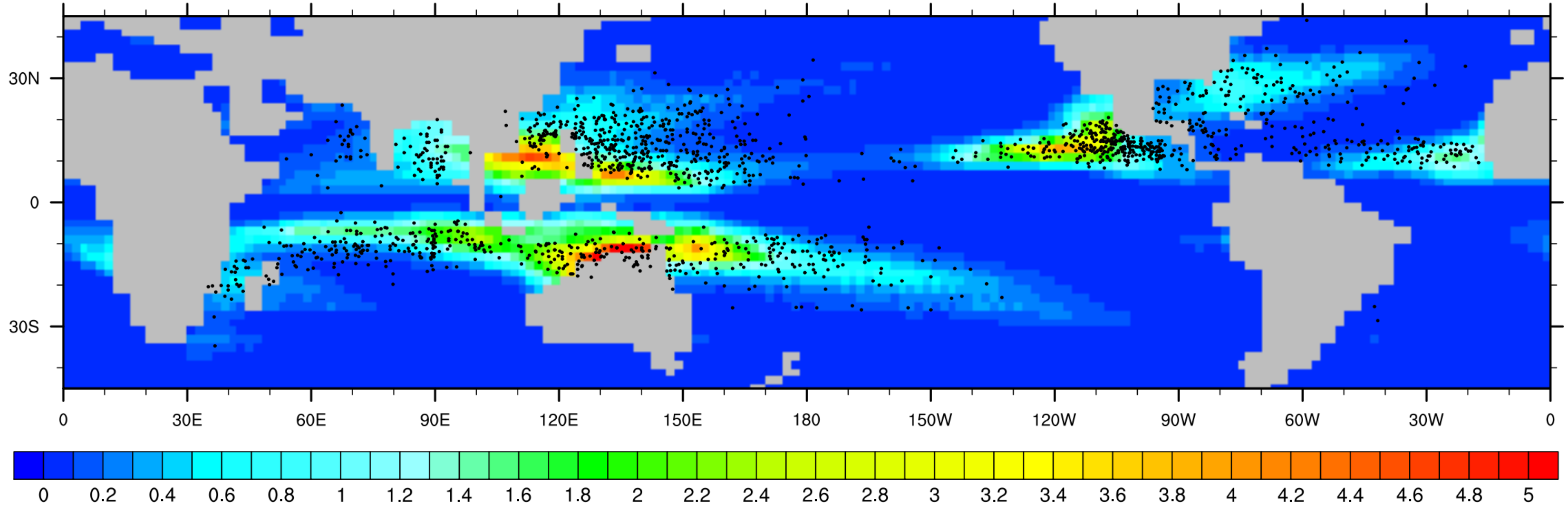

Supplementary Figure 1. Spatial distribution of modern tropical cyclone genesis. Observations (dots) from the International Best Track Archive for Climate Stewardship and the simulation from the NorESM-L based on genesis potential index (shading; $\times 10^{-13}$ m^{-2} $month^{-1}$).

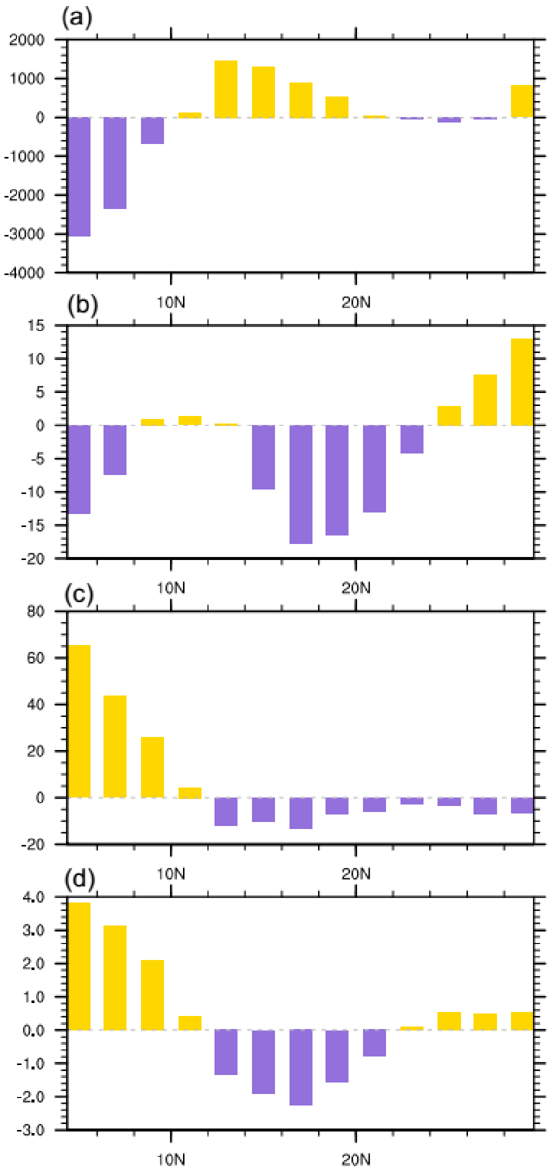

Supplementary Figure 2. Differences in environmental factors over the western North Pacific between the Early Eocene and pre-industrial. (a) Enthalpy difference between sea surface and boundary layer (J kg^{-1}; air-sea disequilibrium) during the storm season, (b) Net radiative flux at the sea surface (W m^{-2}), (c) The convergence of upper ocean heat flux (W m^{-2}; estimated as a residual), and (d) Surface wind speed (m s^{-1}).

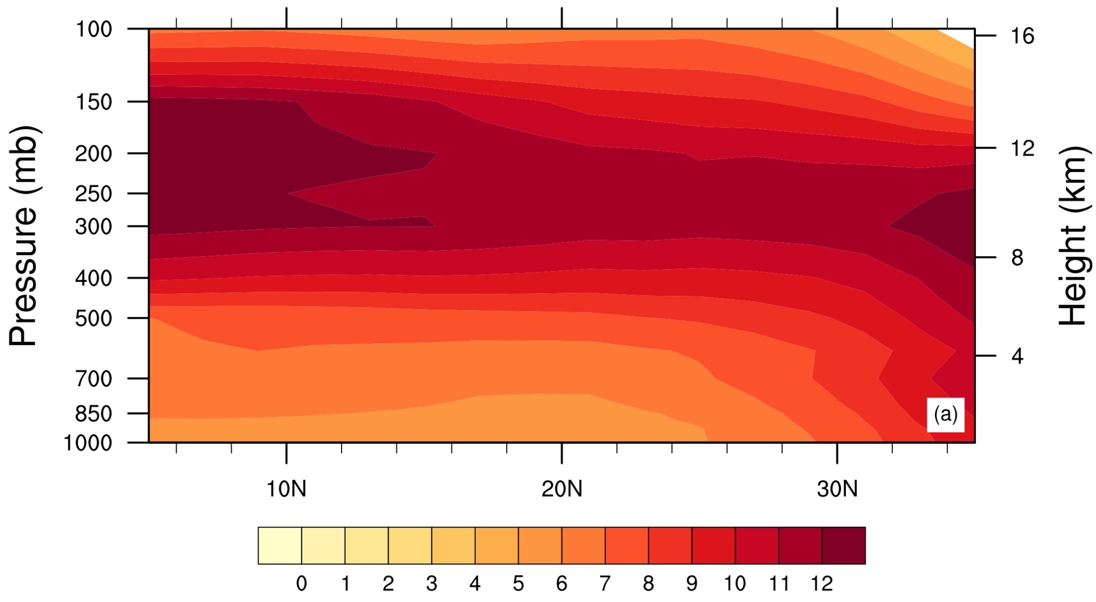

Supplementary Figure 3. Latitude–height cross section of temperature anomaly (ºC) between the Early Eocene and the pre-industrial. Temperature anomaly is averaged over the western North Pacific (110–160ºE) during storm season.

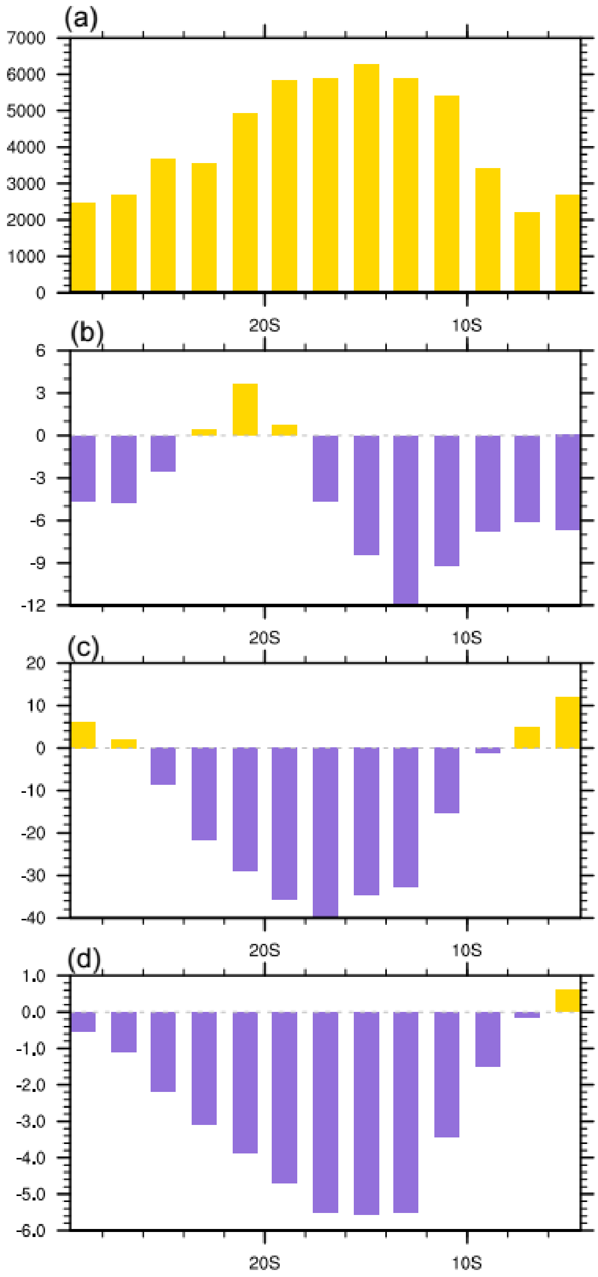

Supplementary Figure 4. Differences in environmental factors over the South Indian Ocean between the Early Eocene and pre-industrial. (a) Enthalpy difference between sea surface and boundary layer (J kg^{-1}; air-sea disequilibrium) during storm season, (b) Net radiative flux at the sea surface (W m^{-2}), (c) The convergence of upper ocean heat flux (W m^{-2}; estimated as a residual), and (d) Surface wind speed (m s^{-1}).

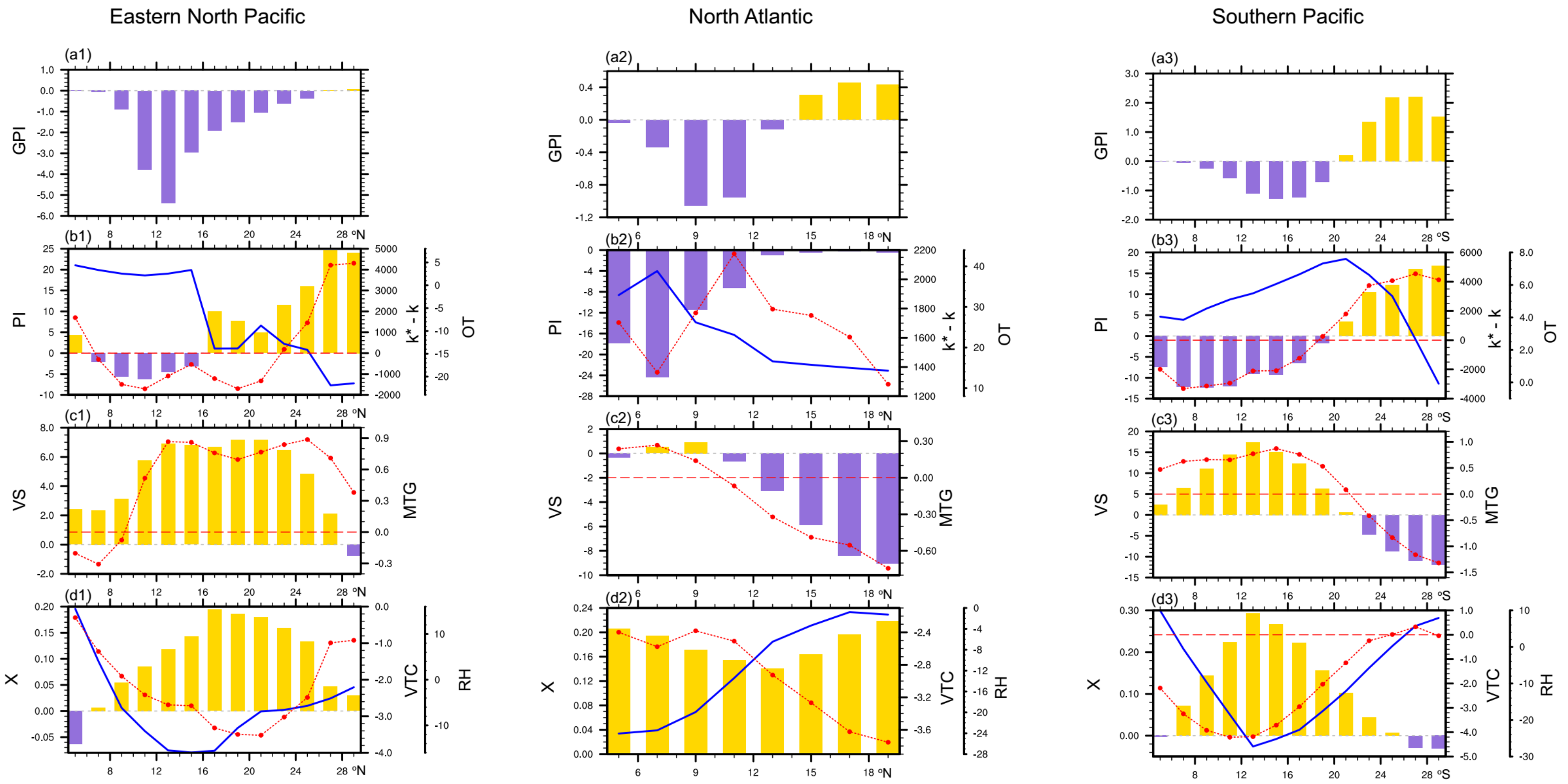

Supplementary Figure 5. Differences in zonal mean genesis potential and environmental variables during storm season between the Early Eocene and pre-industrial. Over the eastern North Pacific (a1–d1; 50–95ºW): (a1) Genesis potential index (GPI; number of events per month). (b1) Potential intensity (PI; bars; m s^{-1}), the enthalpy difference between sea surface and boundary layer (k*–k; red line; J kg^{-1}), and the outflow temperature (OT; blue line; ºC). (c1) Vertical wind shear between 200 and 850 hPa (VS; bars; m s^{-1}) and absolute meridional temperature gradient (MTG; red line; $\times 10^{-6}$ ºC m^{-1}) in the troposphere. (d1) Moist entropy deficit (X; bars), vertical temperature contrast (VTC; red line; ºC) between surface and mid-troposphere (600 hPa), and relative humidity (RH; blue line; %) at 600hPa. (a2–d2) same as (a1–d1) but for the North Atlantic (60–10ºW). (a3–d3) same as (a1–d1) but for the Southern Pacific (165ºE–130ºW).

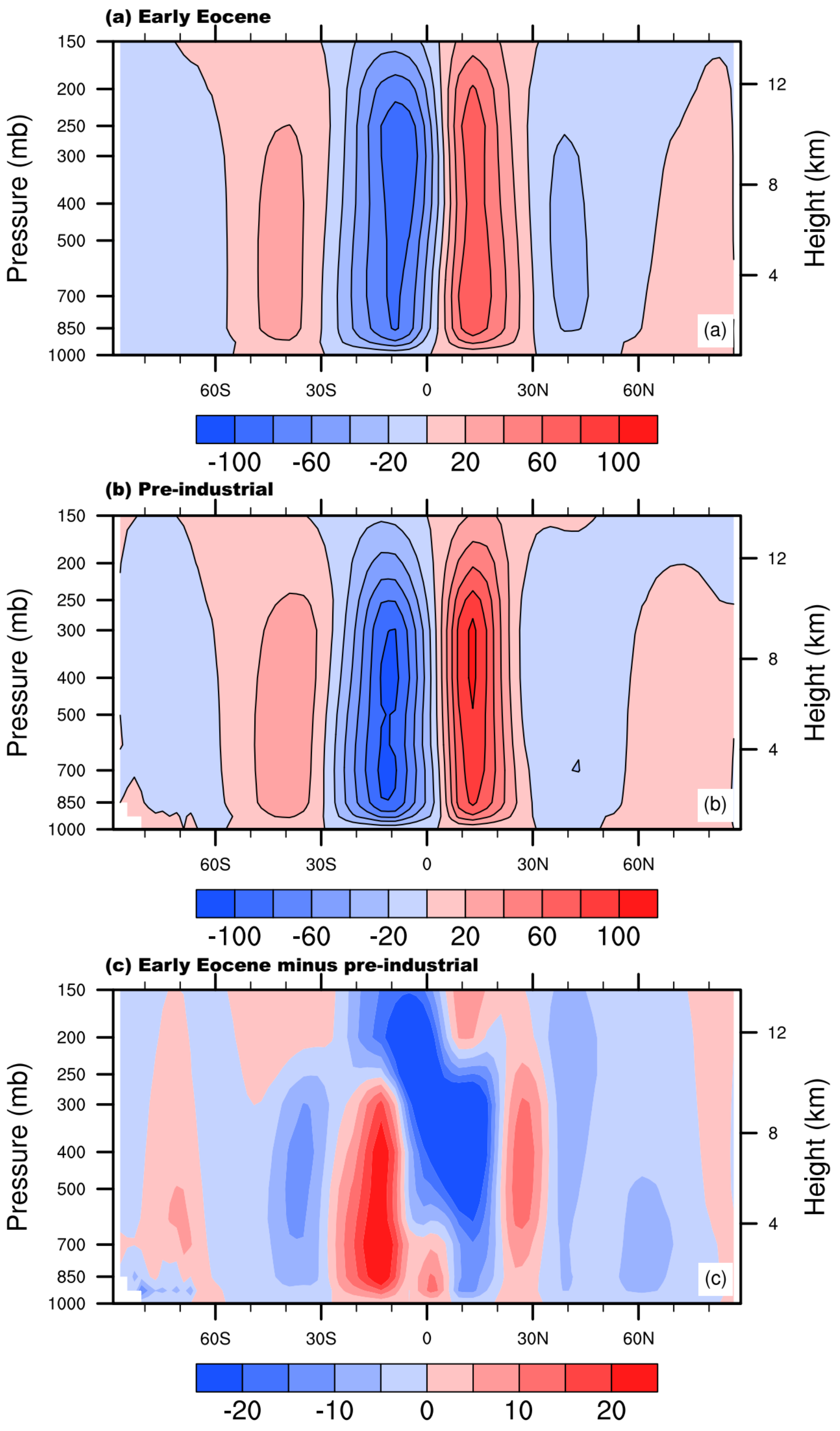

Supplementary Figure 6. The zonal mass stream function. (a) Early Eocene, (b) Pre-industrial, and (c) Difference between the Early Eocene and pre-industrial. The units of mass stream function are $\times 10^9$ kg s^{-1}.

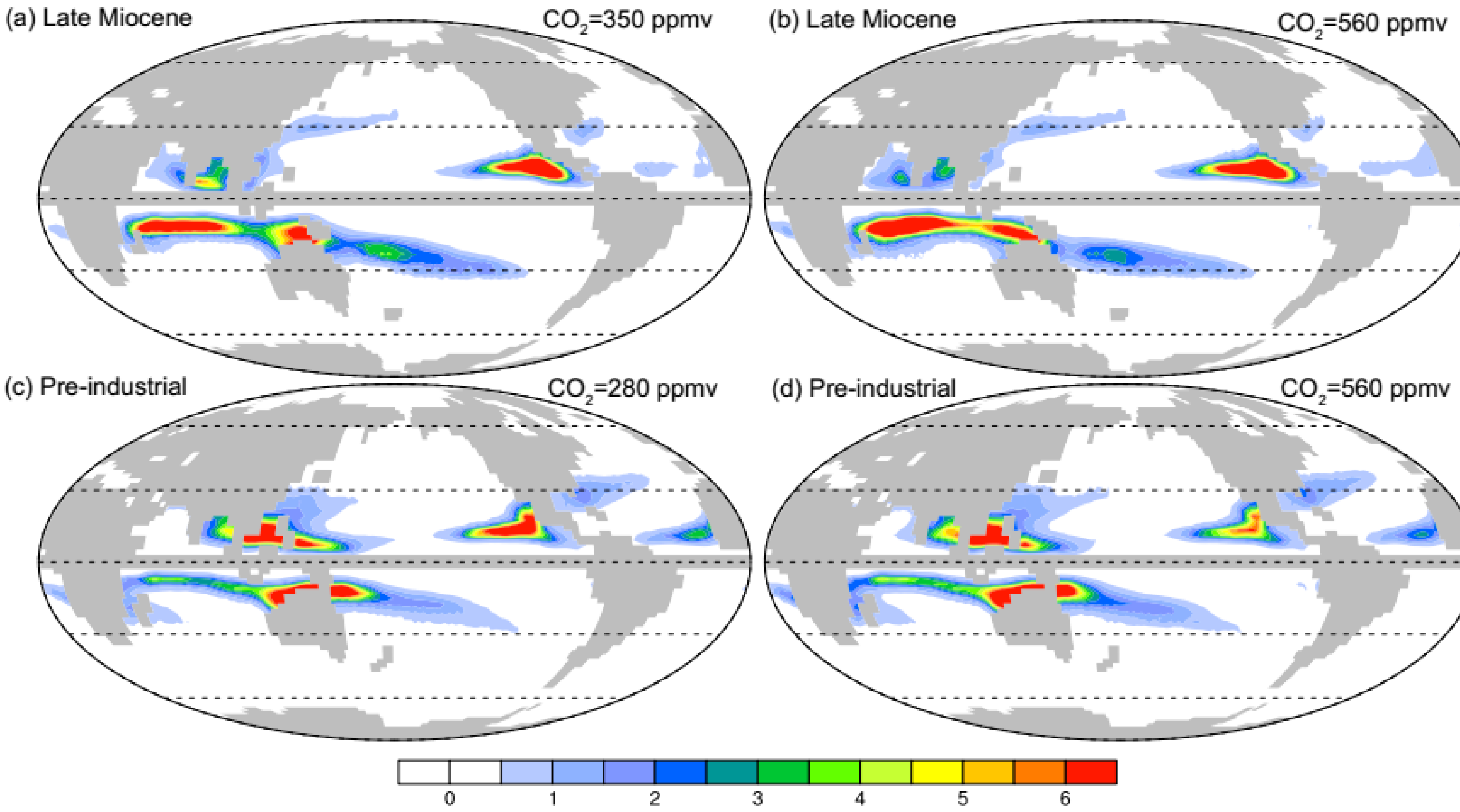

Supplementary Figure 7. Spatial distribution of storm season genesis potential. (a, b) Late Miocene with CO_2 concentration of 350 ppmv (a) and 560 ppmv (b). (c, d) Pre-industrial with CO_2 concentration of 280 ppmv (c) and 560 ppmv (d). The units of genesis potential are $\times 10^{-13}$ m^{-2} $month^{-1}$.

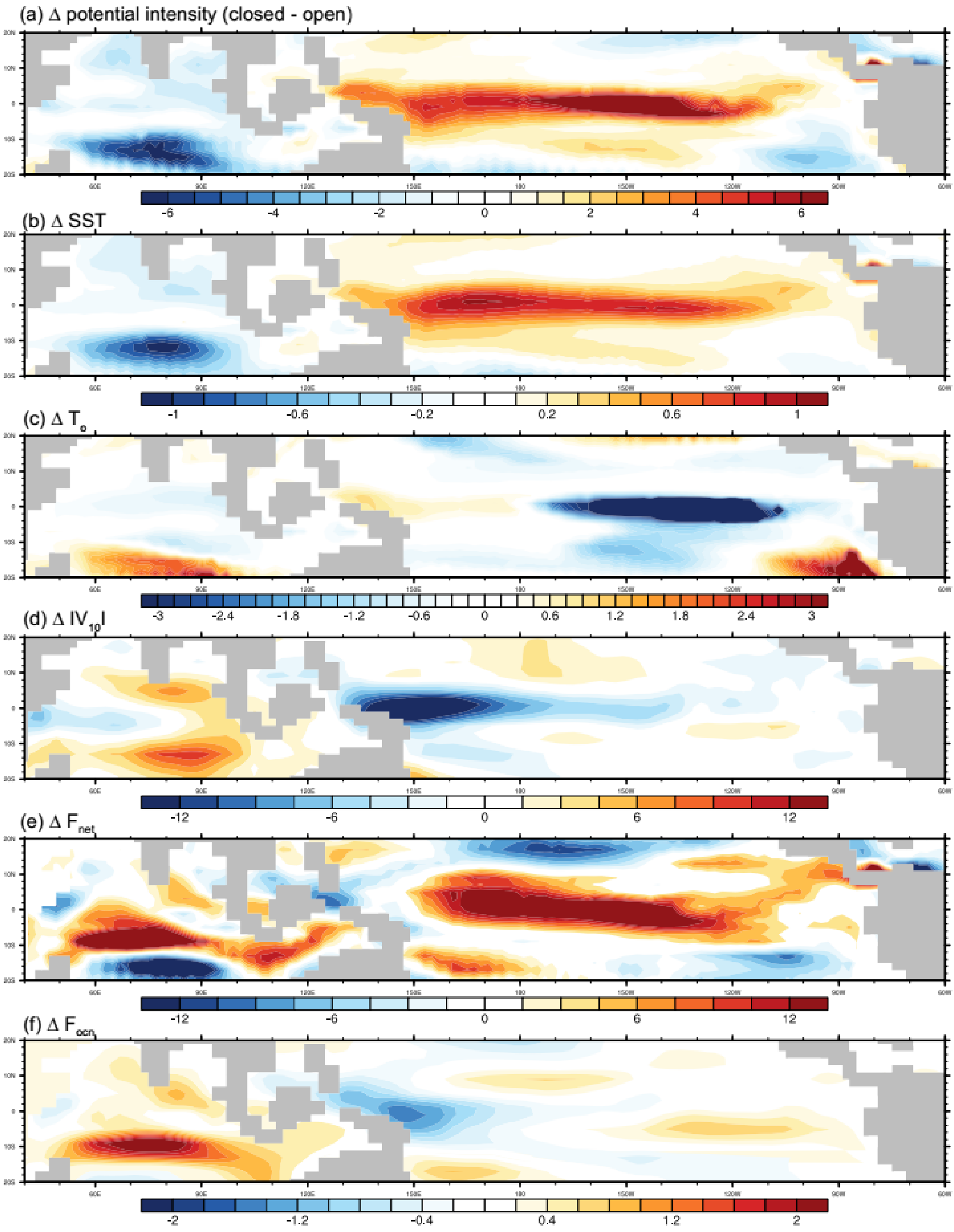

Supplementary Figure 8. Differences in annual mean environmental factors caused by the tropical seaway closures (LP_closeIP minus LP_noclosure). (a) Potential intensity (m s^{-1}), (b) SST (K), (c) Outflow temperature (K), (d) Surface wind speed (m s^{-1}), (e) Net surface radiative fluxes (net downward shortwave radiation minus net upward longwave radiation; W m^{-2}); (f) Ocean heat convergence (positive values indicate anomalous heat convergence; W m^{-2}) calculated as a residual.

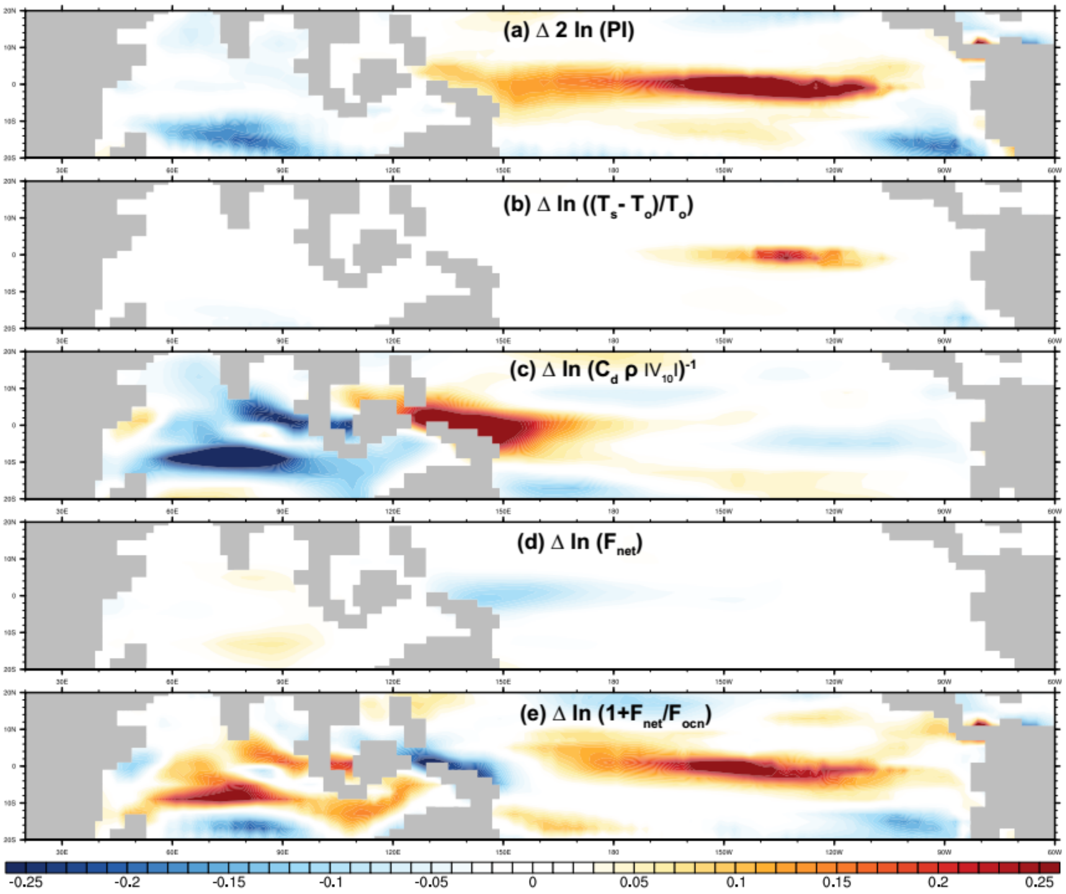

Supplementary Figure 9. Differences in the logarithm of annual mean environmental factors caused by the tropical seaway closures (LP_closeIP minus LP_noclosure). (a) 2*ln (PI), (b) ln ((T_s-T_o)/T_s), (c) ln ($C_d\,\rho\mathbf{V}$)$^{-1}$, (d) ln (F_{net}), and (e) ln (1+ F_{net}/F_{ocn}).

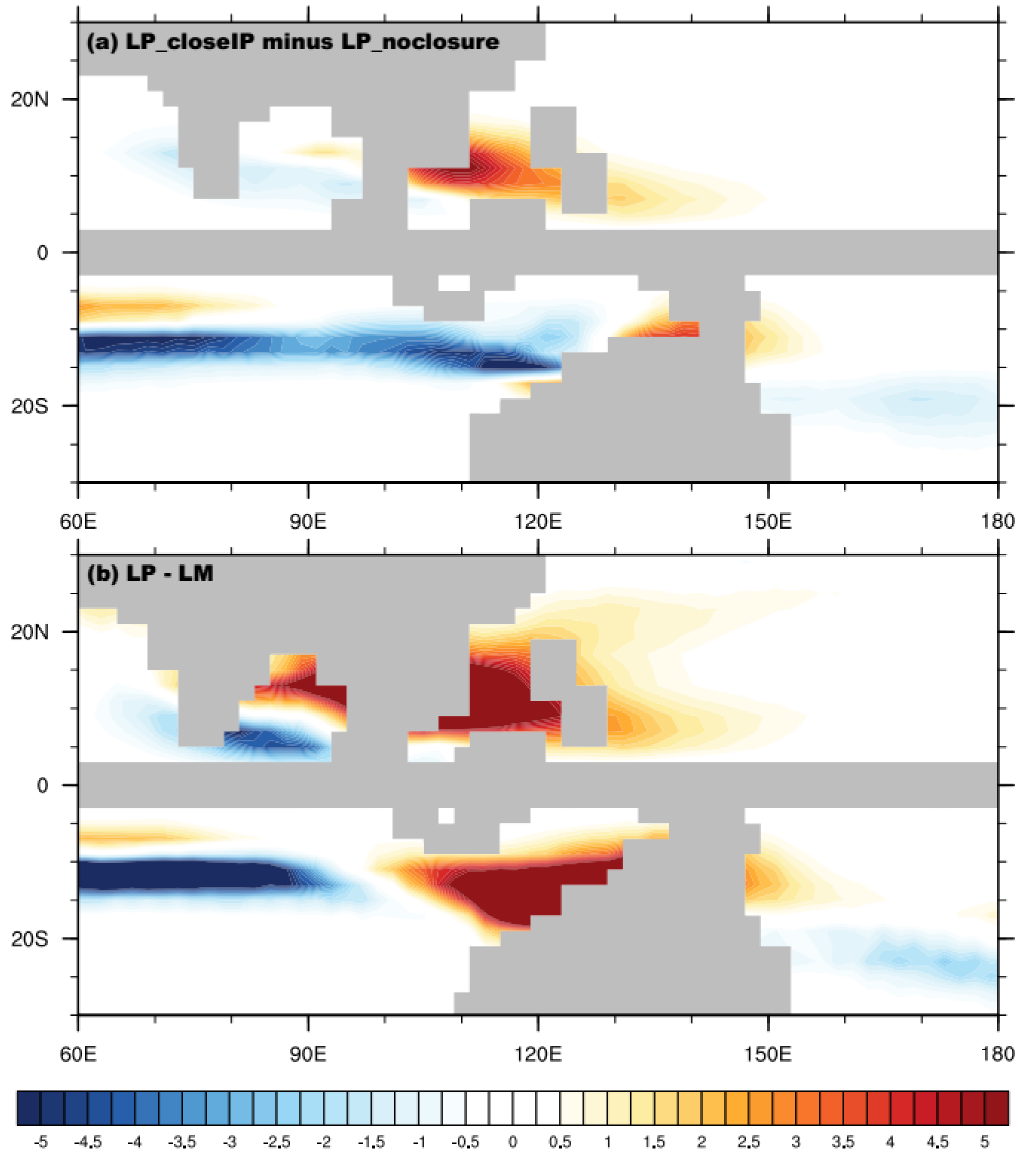

Supplementary Figure 10. Difference in storm season genesis potential. (a) Between the Late Pliocene with closed and opened tropical seaways. (b) Between the Late Pliocene and the Late Miocene. The units of genesis potential are $\times 10^{-13}$ m^{-2} $month^{-1}$.

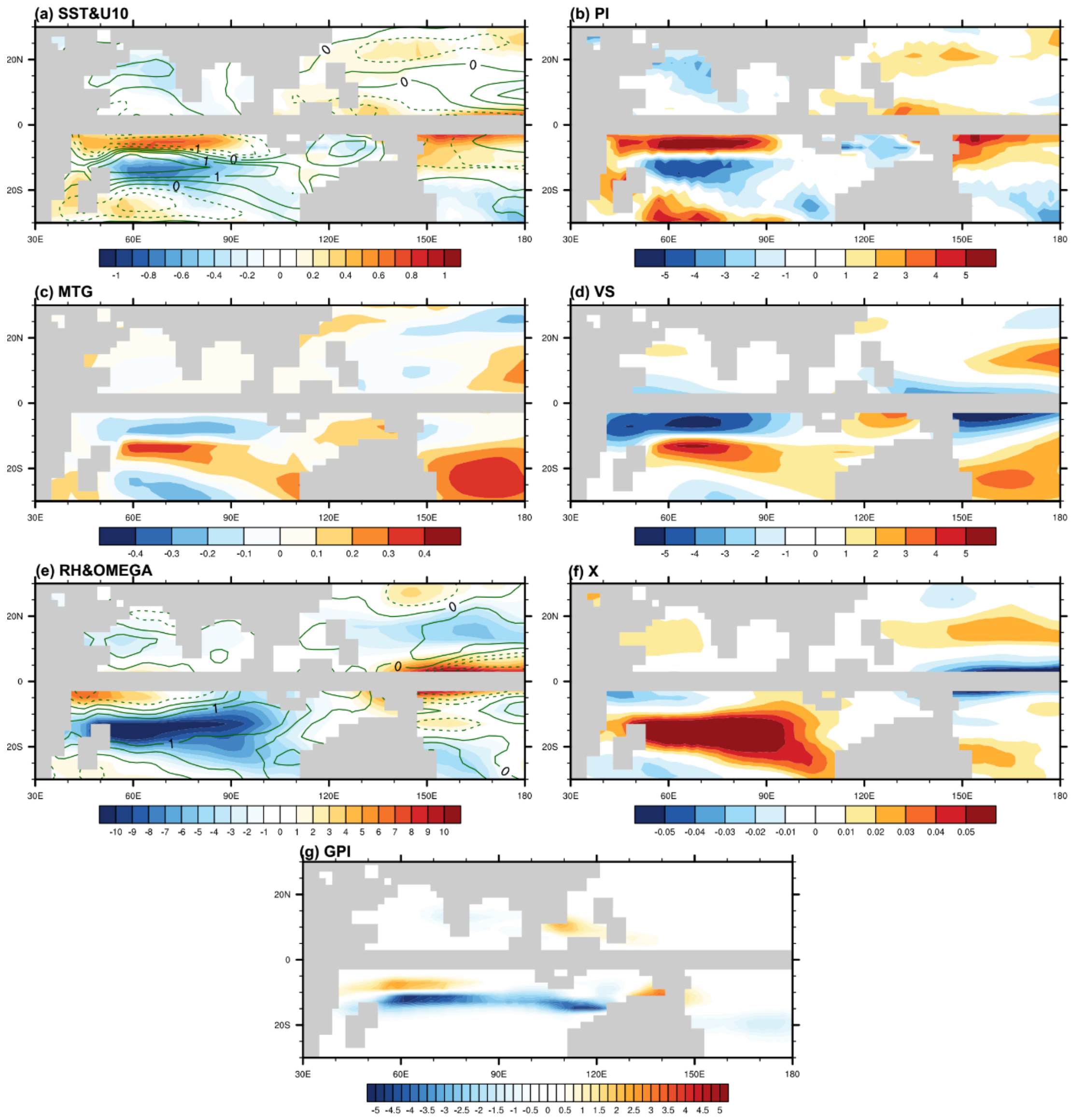

Supplementary Figure 11. Differences in large-scale environmental conditions during storm season caused by the restriction of Indonesia seaway (LP_closeI minus LP_noclosure). (a) Sea surface temperature (shading; ºC) and surface wind speed (contour; m s^{-1}). (b) Potential intensity (m s^{-1}). (c) Absolute meridional temperature gradient ($\times10^{-6}$ ºC m^{-1}) in the troposphere. (d) Vertical wind shear between 200 and 850 hPa (m s^{-1}). (e) Relative humidity (shading; %) and vertical velocity (contour; Pa s^{-1}) at 600hPa. Negative and positive values in vertical velocity show anomalous ascents and descents, respectively. (f) Moist entropy deficit. (g) Genesis potential ($\times10^{-13}$ events m^{-2} $month^{-1}$).

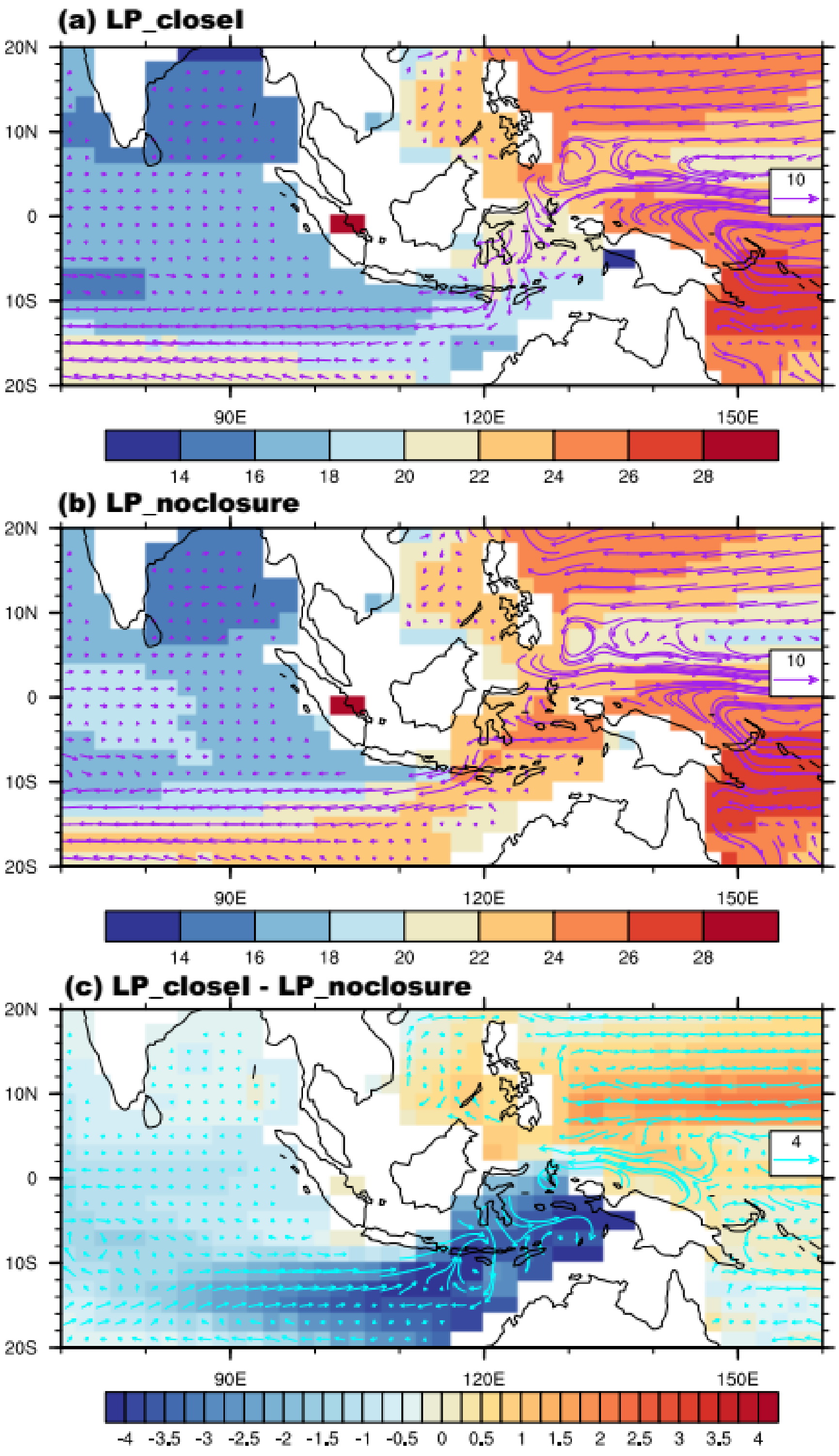

Supplementary Figure 12. Ocean temperatures (shading; °C) and ocean currents (vectors; cm s^{-1}) at 150 m depth. (a) Late Pliocene with the Indonesia seaway closed. (b) Late Pliocene with the Indonesia seaway opened. (c) Difference between the (a) and (b).

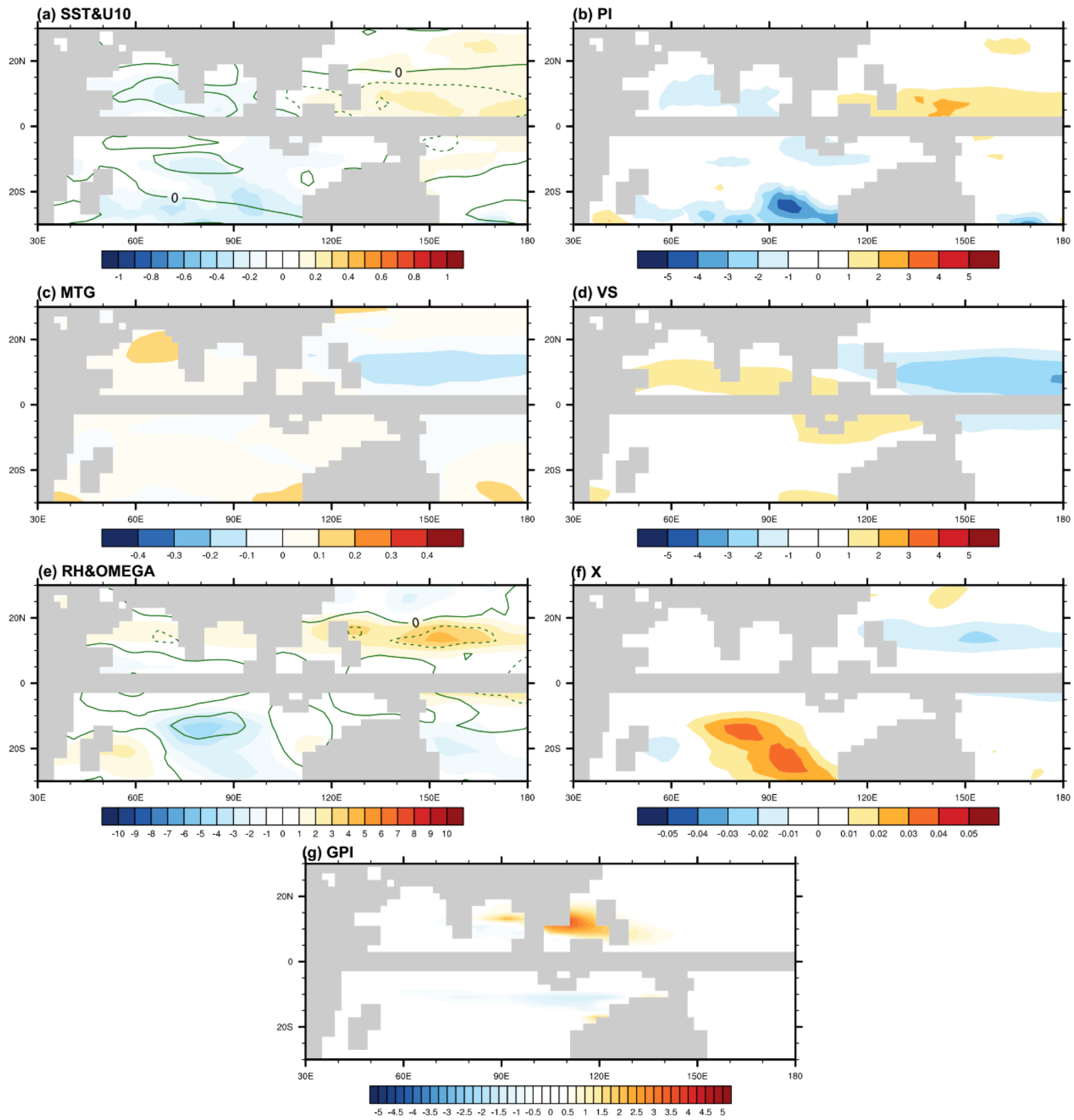

Supplementary Figure 13. Differences in large-scale environmental conditions during storm season caused by the closure of Panama seaway (LP_closeIP minus LP_closeI). (a) Sea surface temperature (shading; ºC) and surface wind speed (contour; m s^{-1}). (b) Potential intensity (m s^{-1}). (c) Absolute meridional temperature gradient ($\times 10^{-6}$ ºC m^{-1}) in the troposphere. (d) Vertical wind shear between 200 and 850 hPa (m s^{-1}). (e) Relative humidity (shading; %) and vertical velocity (contour; Pa s^{-1}) at 600hPa. Negative and positive values in vertical velocity show anomalous ascents and descents, respectively. (f) Moist entropy deficit. (g) Genesis potential ($\times 10^{-13}$ events m^{-2} $month^{-1}$).

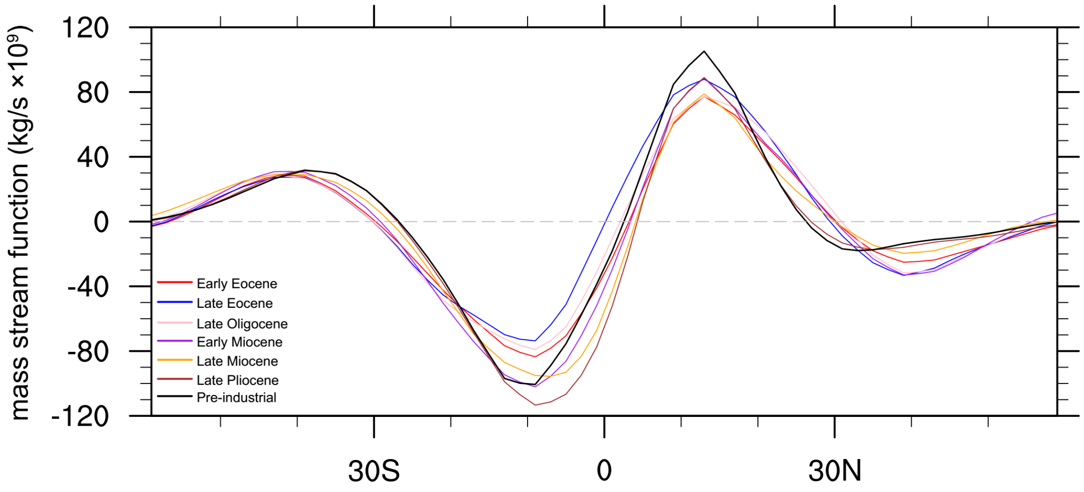

Supplementary Figure 14. Zonal mean of the mass stream function during the Cenozoic. The latitude where the mass stream function becomes zero at the poleward side of the subtropical maximum is defined as the boundary of the Hadley cell.

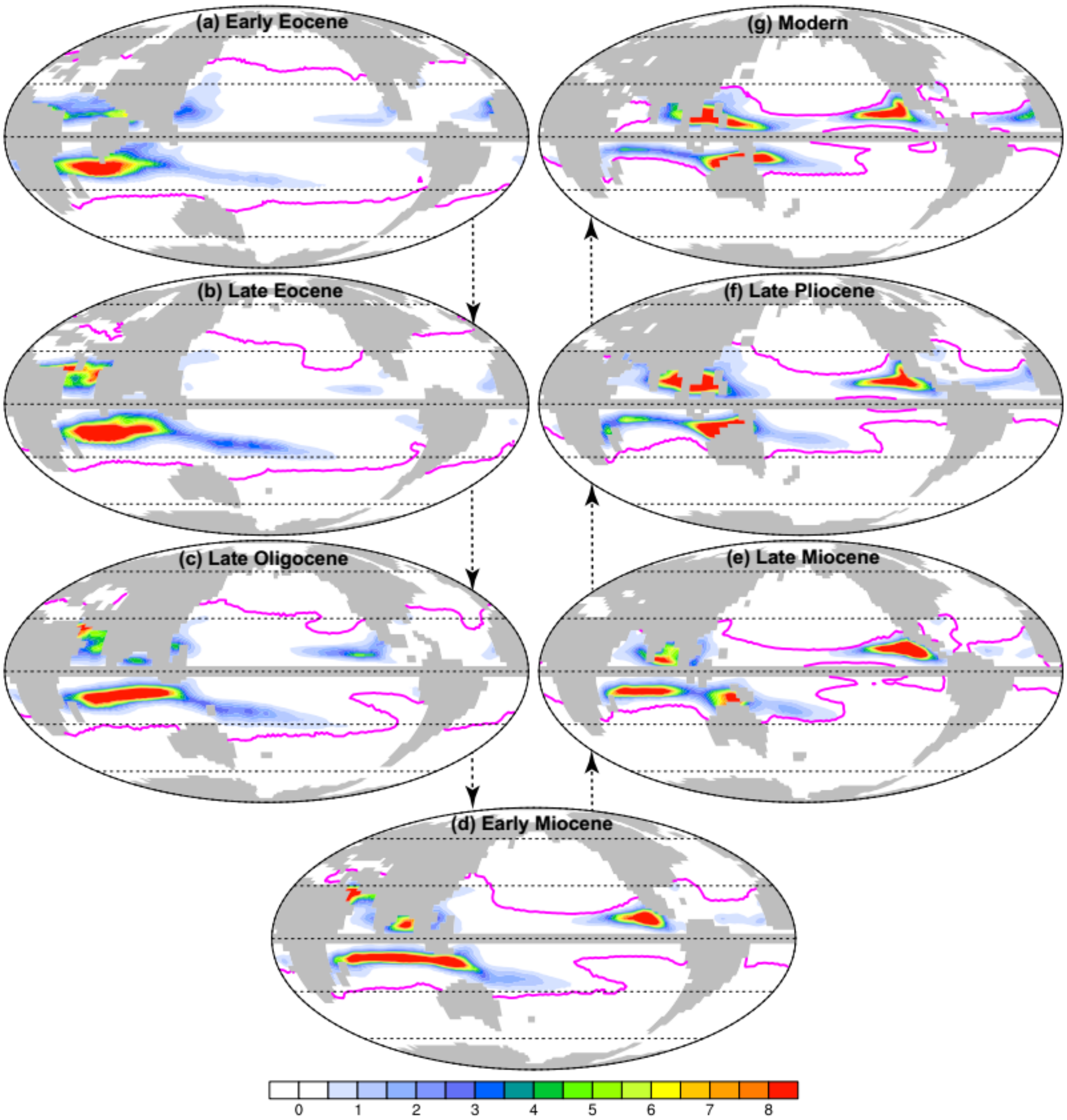

Supplementary Figure 15. Storm season mean genesis potential distribution during the Cenozoic era using the formula developed by Tippett et al. (2011). (a) Early Eocene, (b) Late Eocene, (c) Late Oligocene, (d) Early Miocene, (e) Late Miocene, (f) Late Pliocene, and (g) today. The pink lines show the 26ºC isotherm. The units of genesis potential are events m^{-2} $month^{-1}$($\times 10^{-13}$).

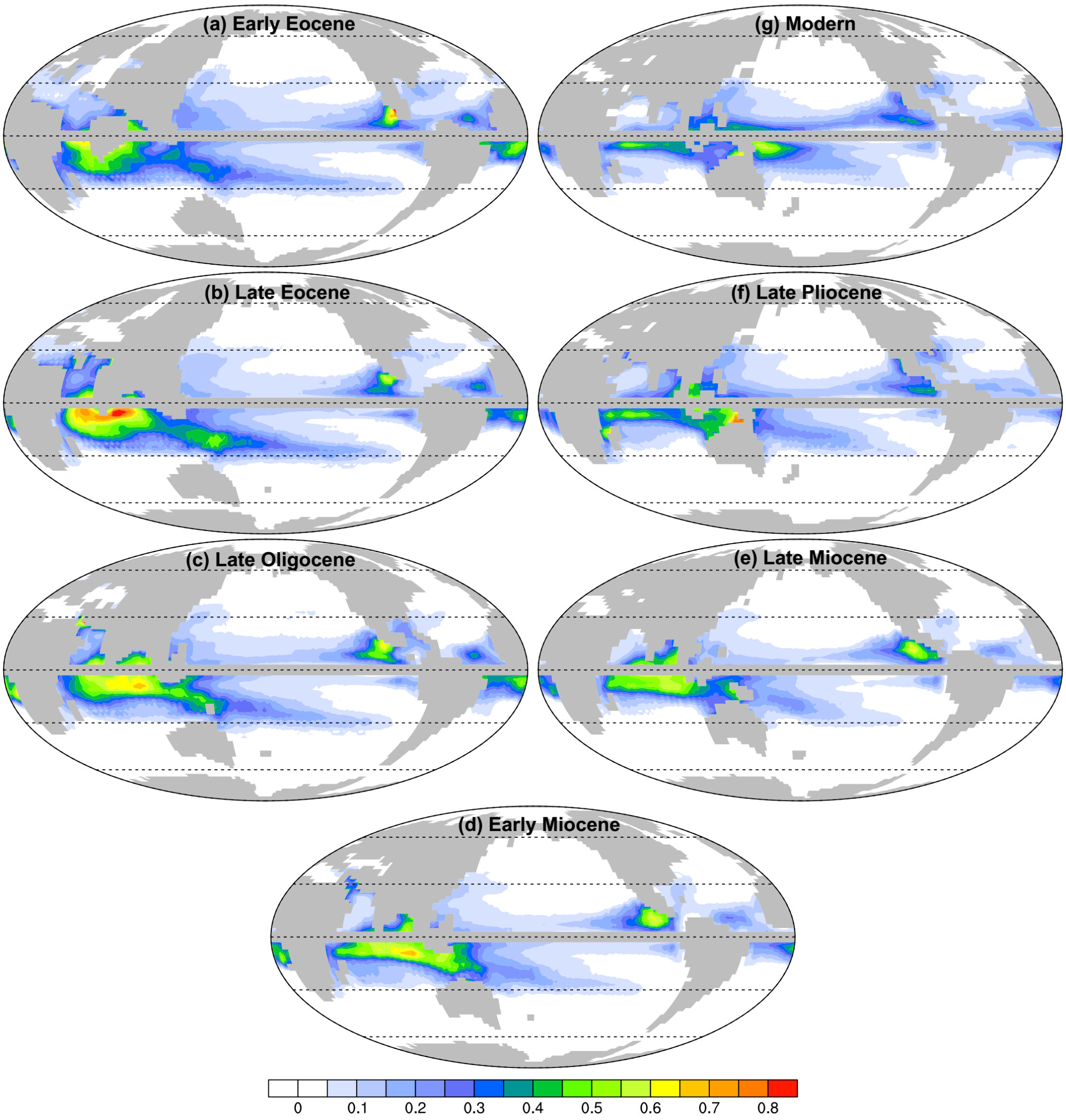

Supplementary Figure 16. Estimated effect of tropical cyclone activity on ocean diffusion coefficient (cm^2 s^{-1}) during the Cenozoic era using the methods of Korty et al. (2008). (a) Early Eocene, (b) Late Eocene, (c) Late Oligocene, (d) Early Miocene, (e) Late Miocene, (f) Late Pliocene, and (g) today. $\kappa_s(x,y)=\kappa\left[\frac{PI(x,y)}{PI_*}\right]^6$, where κ_s is the diffusion coefficient caused by storms, κ is a constant and is set to 0.1 cm^2 s^{-1}, PI is the potential intensity, and PI* is set to 70 m s^{-1} here.

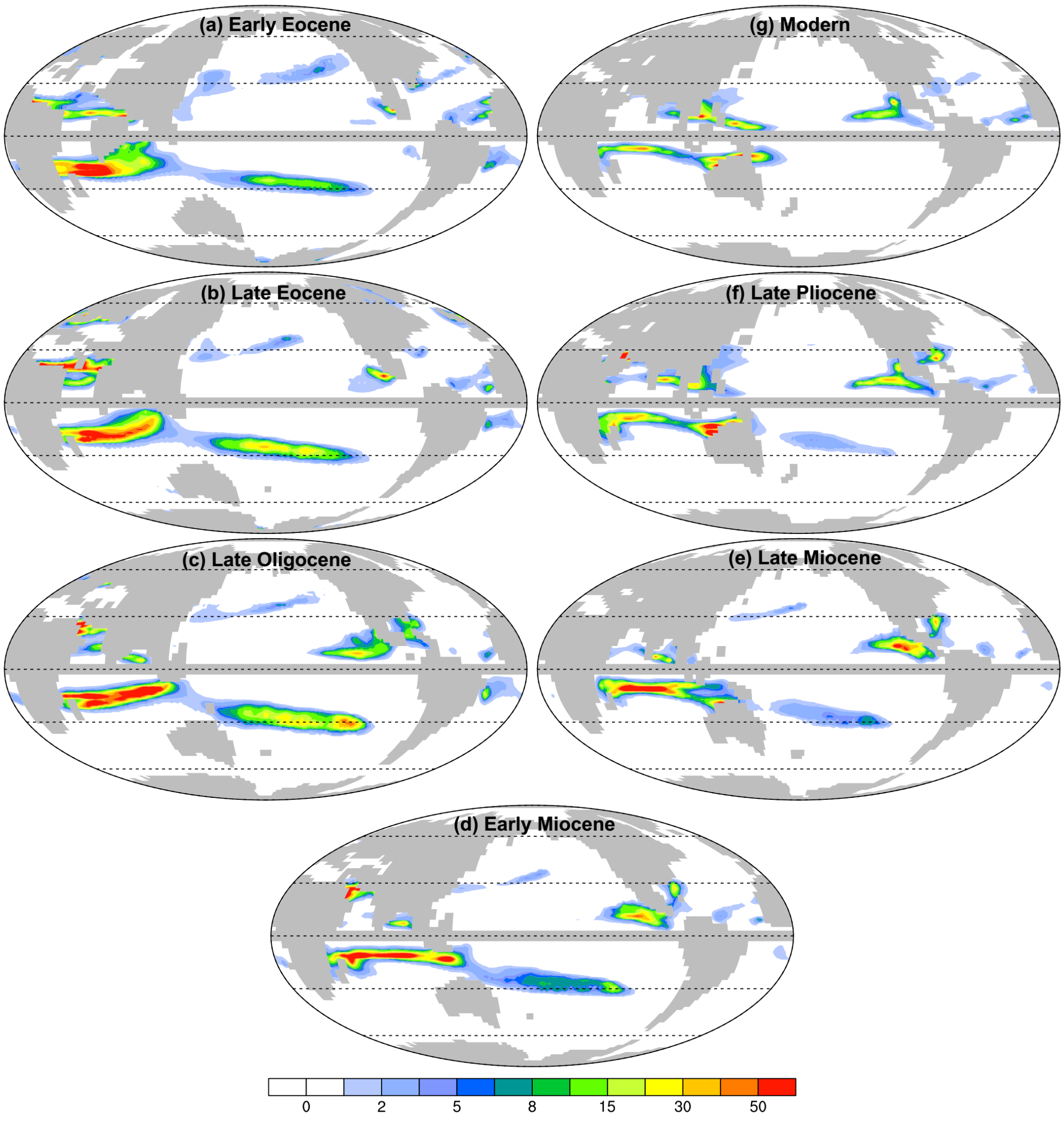

Supplementary Figure 17. Estimated effect of tropical cyclone activity on power dissipation (m^3 s^{-2}) during the Cenozoic era using the methods of Emanuel (2007). (a) Early Eocene, (b) Late Eocene, (c) Late Oligocene, (d) Early Miocene, (e) Late Miocene, (f) Late Pliocene, and (g) today. $PDI \sim |\eta|^{5/2} PI^7 (1+0.3VS)^{-4}$, where PI is the potential intensity, VS is the vertical wind shear between 200 and 850 hPa, and $|\eta|$ is the absolute vorticity at 850 hPa.

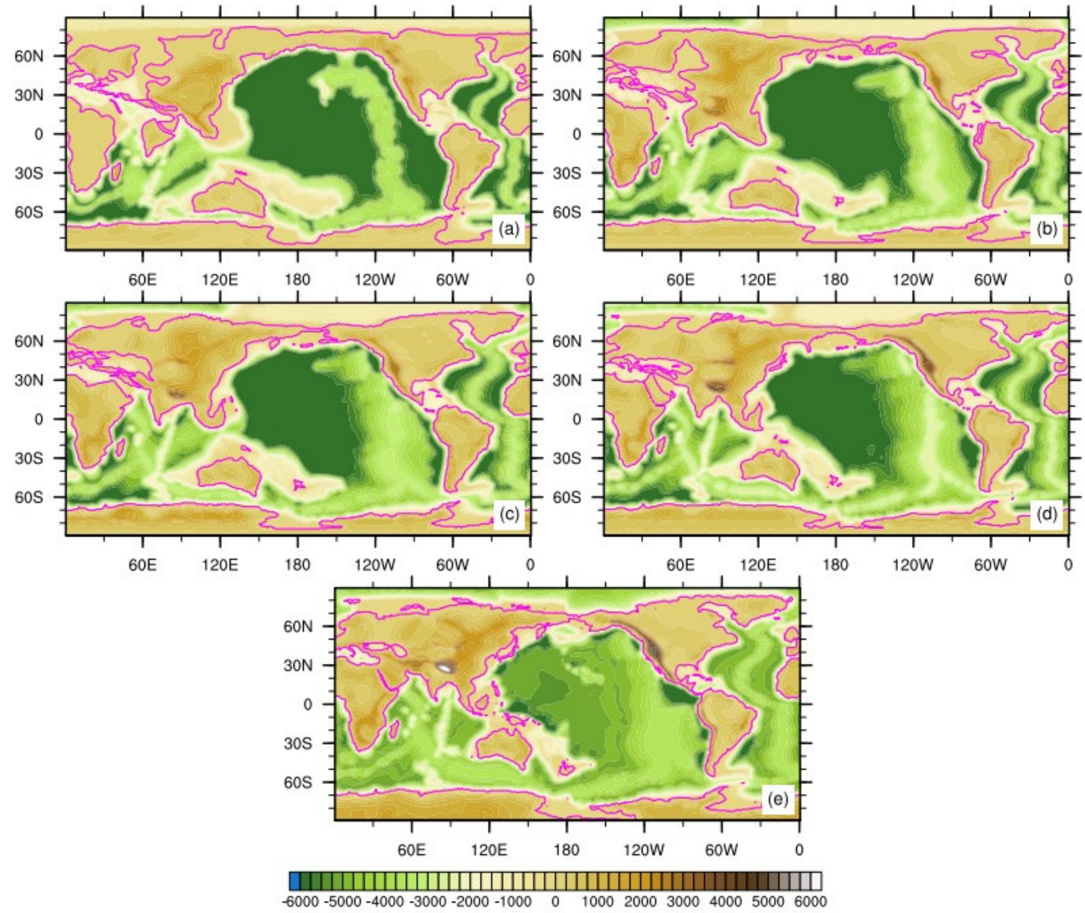

Supplementary Figure 18. Topography (m) used in the NorESM-L model. (a) Early Eocene, (b) Late Eocene, (c) Late Oligocene, (d) Early Miocene, and (e) Late Miocene.

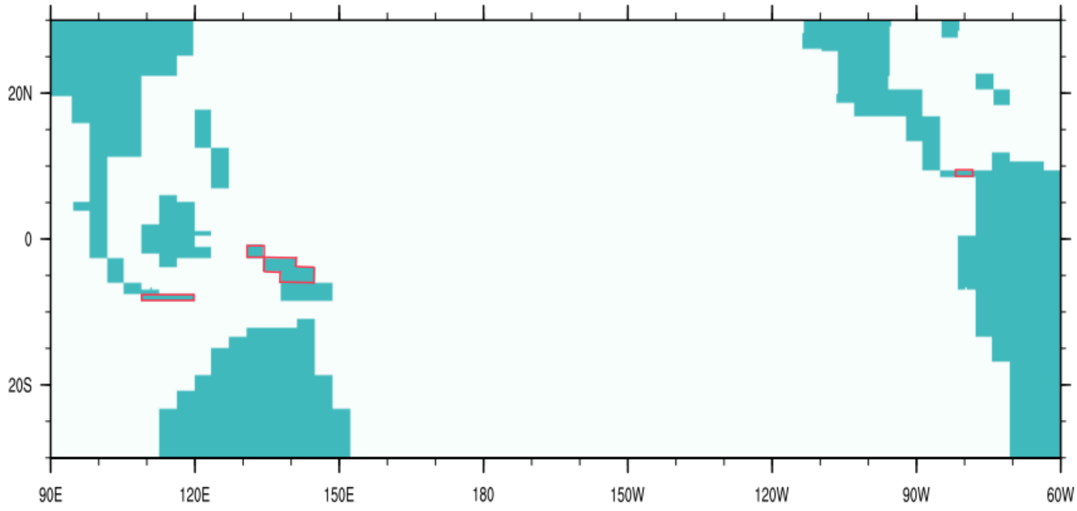

Supplementary Figure 19. Land-sea distribution in the present-day. The grids that are removed in the tropical seaway experiments are highlighted in red rectangular.

Supplementary Tables

Supplementary Table 1. Experimental design for NorESM-L

Exp. ID	CO_2 (ppmv)	Geography	Land-cover	Other conditions
EE	1120	Early Eocene	Idealized*	modern
LE	1050	Late Eocene	Idealized	modern
LO	700	Late Oligocene	Idealized	modern
EM	420	Early Miocene	Idealized	modern
LM	350	Late Miocene	Idealized	modern
LP	405	Late Pliocene#	Late Pliocene	modern
PI	280	modern	modern	modern

*Forest is prescribed between 30ºS and 30ºN, and shrub and grass outside this latitude band without polar ice sheets.

#Land-sea mask is identical to the present-day

Supplementary Table 2. Sensitivity experiments for the tropical seaway closures during the Pliocene

Exp. ID	CO_2 (ppmv)	Topography	Land-sea mask	Land-cover	Other conditions
PI	280	modern	modern	modern	modern
LP_closeIP*	405	Late Pliocene	modern (Indonesia and Panama seaways are closed)	Late Pliocene	modern
LP_closeI	405	Late Pliocene	modern with Panama seaway opened	Late Pliocene	modern
LP_noclosure	405	Late Pliocene	modern with Indonesia and Panama seaways opened	Late Pliocene	modern

*LP_closeIP and LP in Supplementary Table 1 are the same experiment.
